# Contributions to the Development of Fire Detection and Intervention Capabilities Using an Indoor Air Quality IoT Monitoring System

**DOI:** 10.3390/s25206375

**Published:** 2025-10-15

**Authors:** Radu Nicolae Pietraru, Adriana Olteanu, Maximilian Nicolae, Robert-Alexandru Crăciun

**Affiliations:** Faculty of Automation and Computers, National University of Science and Technology Politehnica Bucharest, 060042 București, Romania; adriana.olteanu@upb.ro (A.O.); max.nicolae@upb.ro (M.N.); robertcraciun28@gmail.com (R.-A.C.)

**Keywords:** fire detection, indoor air quality, IoT monitoring, geospatial dashboard, digital twin, smart campus, environmental quality and resilience

## Abstract

This paper presents a method for functionally extending an IoT indoor air quality monitoring network by adding a cloud-level fire detection logic component. The proposed method does not aim to replace traditional fire detection systems at this stage of research, but to propose a solution for the development of fire detection capabilities and to improve the support provided to firefighting teams by providing a geospatial representation of the building in which a fire occurs. The proposed solution is based on a series of laboratory tests that demonstrated that air quality sensors can successfully detect the effects caused by an ignition event of common materials and can differentiate fire events from other events that can generate false-positive alarms by classic detection systems. The research involved five laboratory combustion tests based on the measurement of temperature, humidity, PM2.5 particle concentration, volatile organic compound index, and nitrogen oxide index. Following the tests, a warning mechanism and geospatial representation were designed using a system with ten IoT sensors to monitor the indoor air quality in a building on our university’s campus.

## 1. Introduction

Ensuring the safety and well-being of people inside buildings depends on two critical factors: effective fire detection [1] and maintaining good indoor air quality [2]. As cities expand and buildings become more technologically advanced, there is a growing demand for smarter, more responsive systems to address these concerns. Fires continue to pose serious risks, often resulting in devastating loss of life and property damage. Despite the proven effectiveness of traditional fire detection systems—such as smoke and heat detectors—for providing early warnings during fire incidents, their widespread adoption remains limited [3], particularly in older buildings and under-resourced infrastructures. Many existing buildings, especially residential and legacy commercial constructions, either lack fire detection systems altogether or use outdated devices that operate only locally, offering no remote monitoring or centralized alerting capabilities. By the time these devices detect a fire, the fire may already be spreading rapidly, reducing the time available for safe evacuation and intervention. This delay highlights the urgent need for more proactive solutions that can detect fire hazards at an earlier stage. Upgrading these old structures to meet modern fire safety standards can be technically and financially challenging, especially when retrofitting involves compliance with stringent regulations, specialized installation, and ongoing maintenance.

At the same time, indoor air quality (IAQ) has become a major focus of modern building design, particularly in smart buildings that prioritize energy efficiency, automation, and occupant well-being. Poor air quality, caused by pollutants, volatile organic compounds (VOCs), carbon monoxide (CO), or inadequate ventilation, has been linked to a range of health concerns, from respiratory issues to cognitive impairment [4]. Beyond its impact on health, air quality monitoring can also play a crucial role in fire prevention. Many combustion-related gases, such as carbon monoxide and hydrogen cyanide, can accumulate long before smoke becomes visible, offering a potential early warning system for fire hazards. By closely monitoring fluctuations in air quality, it is possible to detect unusual patterns that may indicate the early stages of combustion, providing critical time for intervention before a fire escalates. On the other hand, after a fire breaks out, IoT-powered smart building systems are invaluable for managing the situation and reducing risks. These systems provide real-time data that can help emergency responders react faster, while their automated features—like fire suppression activation, ventilation control, and power shutdown—work to contain the spread. Smart evacuation systems can also guide occupants toward the safest exits by tracking heat, smoke, and movement, helping to prevent panic and injuries. Even after the flames are extinguished, IoT systems continue to play a crucial role by monitoring the structural safety, detecting lingering toxins in the air, and offering insights into how the fire started and spread. By making buildings more responsive and adaptive, IoT technology not only improves emergency handling, but also strengthens overall fire safety and recovery efforts.

The rise of the Internet of Things (IoT) presents a unique opportunity to address these challenges in a unified and intelligent way [5]. IoT-based systems leverage real-time data from interconnected sensors to monitor environmental conditions continuously. By integrating air quality monitoring with fire detection capabilities, buildings can be equipped with a comprehensive safety system that not only alerts the occupants to hazardous conditions, but also allows for automated responses, such as shutting down electrical circuits or alerting emergency responders before a fire fully develops. Compared to conventional fire detection methods that rely primarily on single-parameter sensing, such as smoke or heat, IoT-based systems integrate multiple sensor types, and some of them have intelligent decision algorithms, in order to enable faster response times and remote monitoring, and to reduce false alarms, thereby offering significantly enhanced reliability and adaptability in diverse environments [6,7]. This proactive approach can significantly reduce response times, enhance situational awareness, and minimize both the human and financial toll of fire incidents.

Beyond safety, these integrated systems align with the broader vision of smart building technology—where automation, sustainability, and efficiency work together to create healthier, safer, and more adaptive living and working environments. Traditional fire detection methods [8] often operate in isolation, but combining them with air quality monitoring allows for a multi-layered defense system that not only detects fires earlier but also improves overall air management within buildings. Additionally, by analyzing the long-term data trends, IoT-based monitoring can help facility managers optimize building ventilation, reduce energy consumption, and prevent issues related to air pollution, further enhancing indoor environmental quality.

This paper explores a method for integration of IoT-based indoor air quality monitoring with fire detection systems, analyzing some experiments on fire detection using indoor air quality sensors. It presents an experimental solution for the functional expansion of an IoT system for indoor air quality monitoring by adding a logical fire detection component in buildings. By merging air quality assessment with innovative fire safety technologies, we aim to highlight a smarter, more efficient approach to protecting lives and property. The purpose of this study is not to provide a complete detection system, but to investigate the fundamental feasibility of using IAQ sensors for fire event detection. The following sections will delve into the system’s design, benefits, technical architecture, experiments, and its potential to redefine fire safety standards in the era of smart buildings.

The paper is structured as follows:

- Section 2 presents the fire detection systems and methods found in the scientific literature, and a compares the solution proposed in this paper with those from other studies.
- Section 3 describes the IoT indoor air quality monitoring system that underlies the proposed technical solution. The paper uses this IoT monitoring system to add new fire detection functionality.
- Section 4 presents the experiments on fire detection using the indoor air quality sensors and a statistical analysis of the normal functioning of the sensors to determine the thresholds for detecting abnormal situations.
- Section 5 presents the original solution for implementing the detection logic within the cloud platform of the monitoring system. A valuable component of the solution is the geospatial representation of the interior space affected by a fire, which allows for the planning of the intervention by fire teams.
- Section 6 draws conclusions and outlines future developments.

## 2. Fire Detection in Scientific Literature

Fire detection systems are a crucial part of any building’s safety measures, designed to identify potential fire hazards as early as possible. Their main objective is to alert occupants and emergency responders quickly enough to prevent injuries, loss of life, and property damage. Over the years, these systems have evolved significantly, moving from simple smoke alarms to more advanced IoT-connected technologies that have helped improve both detection speed and accuracy.

Fire detection systems work by sensing different indicators of fire, such as smoke, heat, or gas. The most common types include the following:

- Smoke detectors: These are the most widely used fire detection devices, designed to sense smoke particles in the air.
- Heat detectors: Instead of sensing smoke, these devices detect high temperatures or sudden increases in heat.
- Flame detectors: Using infrared or ultraviolet light sensors, these detectors can identify flames almost instantly, making them useful in industrial settings where fire hazards are high.
- Gas detectors: These detect harmful gases, such as carbon monoxide (CO) and volatile organic compounds (VOCs), which can indicate early combustion before smoke is even visible.
- Smart and IoT-based detectors: These modern systems combine multiple sensors with IoT technology, providing real-time monitoring, automated alerts, and improved accuracy at detecting fire hazards.

Advancements in technology have led to smarter, faster, and more reliable fire detection methods, such as IoT-connected fire detection systems. Smart sensors linked to the internet allow for real-time monitoring, instant mobile alerts, and seamless integration with building automation systems or air quality monitoring for early detection. Instead of waiting for smoke to appear, modern sensors can detect changes in air quality—such as rising levels of CO or VOCs—which may signal early combustion, and so on.

Table 1 compares this study’s differences with the more relevant studies in the literature, although most of these studies had similar objectives (e.g., detection systems, monitoring parameters, decision criteria, connectivity, IoT platform, map/area/intervention, and alarms).

## 3. Indoor Air Quality IoT Monitoring System

**Figure 1:**
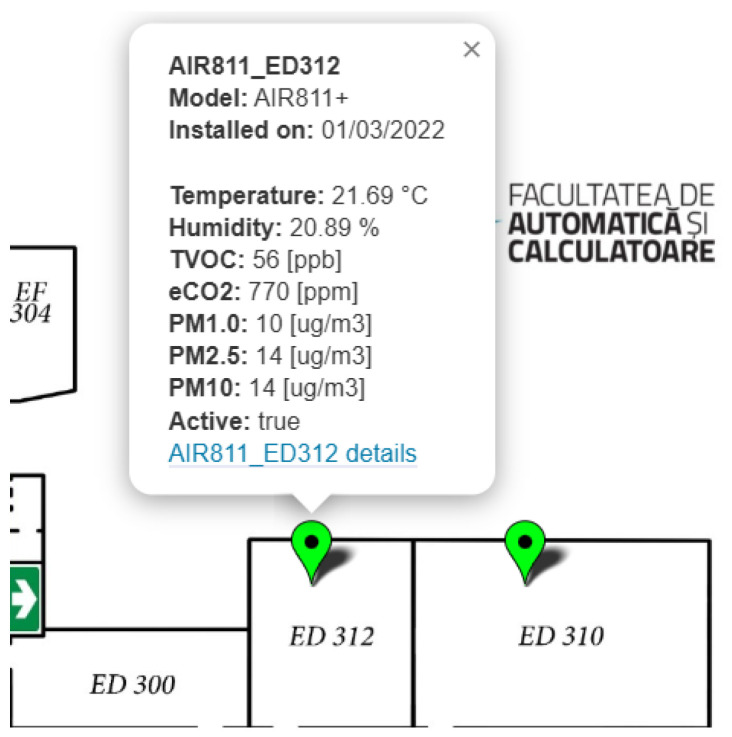
IoT indoor air quality monitoring dashboard (fragment with detail for the AIR811_ED312 sensor) for the first two laboratory rooms on campus (ED312 and ED310)—early 2022 (the green color of the sensors on the map indicates excellent indoor air quality).

The solution proposed in this paper is based on an IoT platform that already exists in three buildings of the National University of Science and Technology Politehnica Bucharest campus. The purpose of this section is to present this platform in order to determine the advantages of implementing the new method resulting from the current research. The IoT platform for monitoring indoor air quality began to be developed in early 2022 at the end of the isolation period generated by the COVID-19 pandemic. The resumption of indoor activities on the university’s campus was performed under strict constraints related to the prevention of the spread of diseases; for this reason, an attempt was made to implement automatic measures to monitor the risk of disease transmission generated by non-compliance with social distancing rules. Based on research [18] conducted during the isolation period, an air quality monitoring method was implemented in two laboratories at the Faculty of Automation and Computers, within Politehnica Bucharest, to determine the number of people in a laboratory and to signal cases of overcrowding associated with an increased risk of spreading airborne diseases [Figure 1].

The monitoring platform has been active without interruption since the beginning of 2022, and is used for experimental scientific studies (related to ways to quickly implement an IoT monitoring network [17] and data processing [19]) as well as an example for students as a method of implementing an IoT platform. Now, the platform integrates three different buildings and 40 IoT sensors from various research projects; the last 10 sensors were added specifically for the research presented in this paper. The IoT monitoring devices are heterogeneous in terms of monitored parameters (noise level, light intensity, number of people in a room, ambient temperature and humidity, dust concentration in the air, concentration levels of volatile organic compounds (VOCs) and nitrogen oxides (Nox)) and communication methods (WiFi and LoRaWAN). The 10 sensors used for the tests in this research communicate via WiFi with the IoT platform and record the following parameters: ambient temperature and humidity, concentrations of particles in the air (PM1.0, PM2.5, PM4.0, and PM10), and index level of volatile organic compounds (VOCs) and nitrogen oxides (Nox).

The IoT air quality monitoring sensors used in the current research are based on NodeMCU development boards, which utilize an Espressif (Espressif Systems, 690 Bibo Road, Shanghai, China), ESP8266 WiFi microprocessor and Sensirion (Sensirion AG, Laubisruetistrasse 50, 8712 Stäfa, Switzerland) SEN55 air quality sensors. The Sensirion SEN55 digital sensor is a low-cost integrated sensor capable of measuring nine environmental parameters (temperature; humidity; VOC Index; NOx Index; and concentration of particles in the air with a diameter of less than 1.0, 2.5, 4.0, and 10 μm—for PM10 it provides their mass and numerical concentration). Details of the sensor’s measurement performance are presented in Table 2. The sensor has a lifespan of over 10 years and is a very good option for HVAC devices and also for indoor air quality monitoring devices, simplifying the design process and integration into new devices.

**Figure 2:**
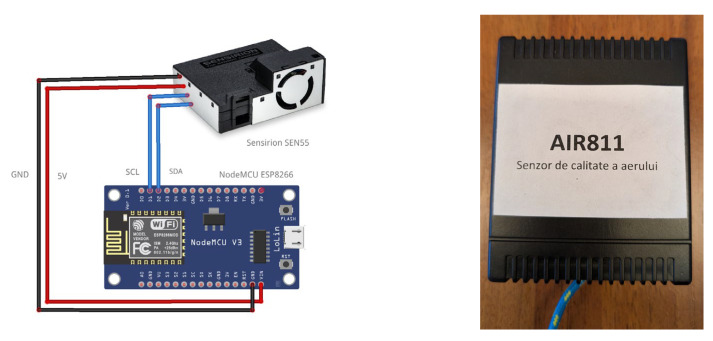
IoT monitoring device ((**left**)—schematics; (**right**)—real device).

**Figure 3:**
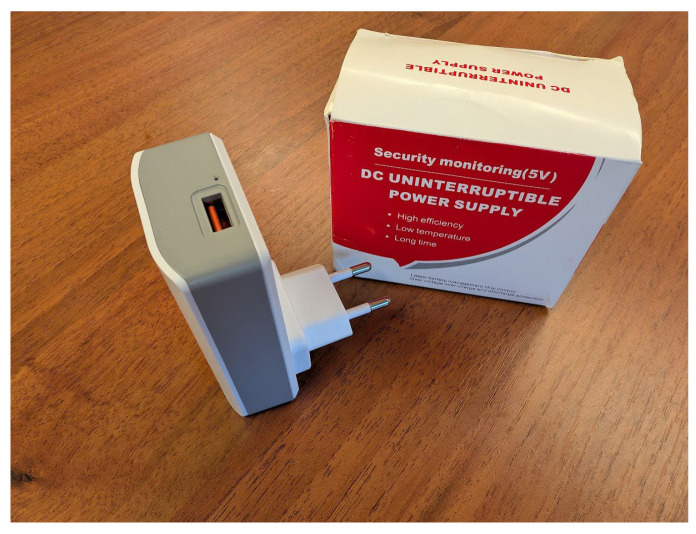
Uninterruptible power supply for AIR811 sensors: input, 220 V/0.5 A; output 5 V/2 A. Sensor operation time up to 30 min (measured in practical testing).

The connection between the sensor and the development board is achieved via the I2C protocol (Figure 2. The system can be powered by a 5 V/1 A mains power supply, but to ensure power independence in emergency situations, the AIR811 sensors are powered by a QX-2152B uninterruptible power supply (Figure 3).

**Figure 4:**
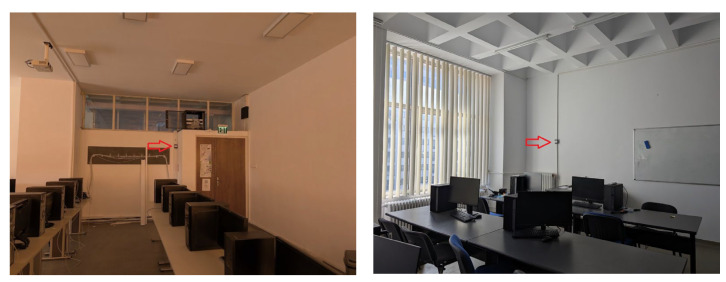
IoT monitoring devices installation examples (left room, ED310; right room, ED312—devices identified by the red arrow).

As shown in paper [17], the monitoring solution is a low-cost monitoring solution with a quick and economical installation. The IoT sensors use the university’s WiFi infrastructure to communicate with an IoT server that records, processes, and displays the information to users. The university’s WiFi infrastructure is based on a mesh architecture providing coverage from multiple AP devices for all areas for redundancy. The AP devices are powered by a dedicated power network that is connected to the campus’ emergency generators, ensuring continuity in case of emergency situations. Communication between the IoT monitoring sensors and the server is achieved via the MQTT protocol. The IoT monitoring sensors are installed vertically on walls at a height of approximately 1.80 m from the floor (as can be seen in the images in Figure 4).

**Figure 5:**
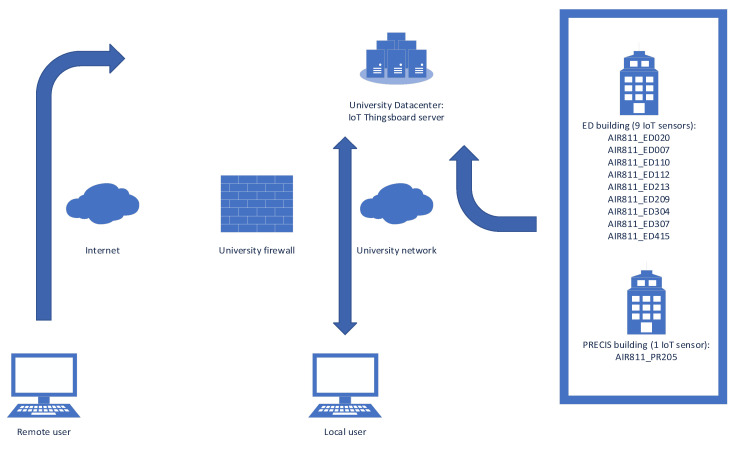
IoT monitoring system architecture.

The central IoT server is hosted in the university’s data center as a virtual machine and runs CentOS Stream Linux 8. The IoT functionality is provided by the open-source ThingsBoard Community Edition version 3.8.1 platform. The data were saved in a PostgreSQL database version 12.22. Communication between the IoT monitoring systems is carried out exclusively via the university’s local network; the architecture of the monitoring system is presented in Figure 5. The IoT device management interface and data visualization can be performed both locally via the university’s network and remotely via the internet network using a web browser.

## 4. Experiments on Fire Detection Using Indoor Air Quality Sensor

The experiments conducted in this research aimed to verify the possibility of detecting fire-type events using the indoor air quality monitoring device presented in the previous section. Nine of the IoT monitoring devices were installed in the ED building on the university’s campus to monitor laboratory rooms where teaching activities are carried out normally, and the tenth device was installed in a research laboratory in the PRECIS building and was used to conduct experiments.

The experiments consisted of five tests (Table 3). All five tests consisted of placing an AIR811_PR205 sensor in an isolated enclosure measuring 30 cm × 50 cm × 55 cm, in which the events were triggered in a controlled manner. Three events were fire-type and involved the ignition of small quantities (10–20 g) of stickers (test 1), wooden matches (test 2), and plastic (test 3) in the same isolated enclosure as the IoT device. The materials that were set on fire were chosen to mimic as closely as possible the mixture of materials found in a typical laboratory room. The other two events were events that would normally falsely trigger a fire alarm, and consisted of generating a large amount of steam (using an electric teapot, test 4) and a large amount of dust (by reversing the ventilation direction of a dirty vacuum cleaner, test 5).

Each of the five tests lasted 10 min, followed by at least 10 min of ventilation of the test enclosure. For each test, the IoT monitoring platform recorded the data measured by the Sensirion SEN55 sensor.

The methodology for conducting the experiments was deliberately designed in the simplest possible manner to ensure repeatability and to prove that IAQ sensor networks have the sensitivity to capture the signals of an incipient fire. In addition to the tests, a statistical analysis was also performed on the evolution of the measured parameters under normal conditions. The data collected over three months were analyzed to establish a variation threshold for normal conditions for a laboratory on the university’s campus.

### 4.1. Test 1—Description, Execution, and Data

**Figure 6:**
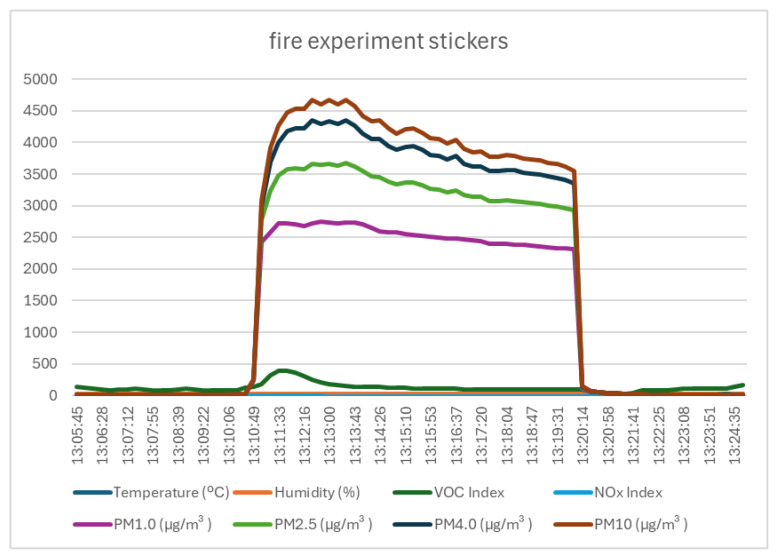
Graph of data recorded by monitoring system in case of sticker burning.

The first test consisted of igniting small pieces of stickers. The stickers were considered to contain a combination of paper, ink, laminated plastic, and glue, elements that are found in books, technical documentation, and other teaching materials normally present in a laboratory room and that would easily ignite in the event of a fire. The results recorded using the SEN55 sensor (shown in Figure 6 and Table 4) during the first test showed insignificant variations in the temperature, humidity, and NOx index parameters, and strong variations in all the dust particle sizes (PM1.0, PM2.5, PM4.0, and PM10) and the VOC index. The VOC index varied during the test by 364 units, i.e., 72% of the measurement range of 500 units. The concentration of particles in the air varied by over 2000 µg/m^3^ (in the case of PM1.0) and over 4000 µg/m^3^ (in the case of PM10). The variation in particle concentrations was observed to be the strongest response recorded following the ignition of the test materials, which was easily explained by the appearance of smoke generated by the fire.

### 4.2. Test 2—Description, Execution, and Data

**Figure 7:**
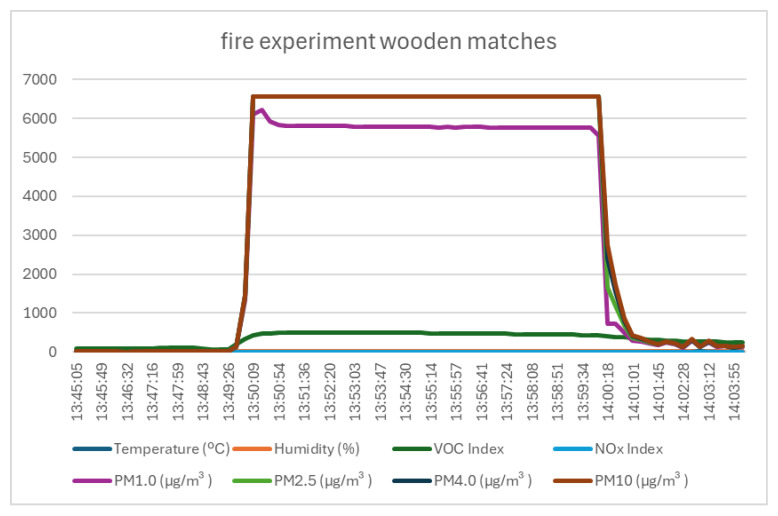
Graph of data recorded by monitoring system in case of wooden matches burning.

The second test aimed to simulate the ignition of a common material used in the manufacture of modern furniture—processed and treated wood. For this, wooden matches were used. As in the previous test, no major variations were recorded for the temperature, humidity, or nitrogen oxides, but only for the volatile organic compounds (variation of over 300 units) and dust particles (variations between 2700 µg/m^3^ for PM1.0 and 4670 µg/m^3^ for PM10)—as shown in Figure 7 and Table 5.

### 4.3. Test 3—Description, Execution, and Data

**Figure 8:**
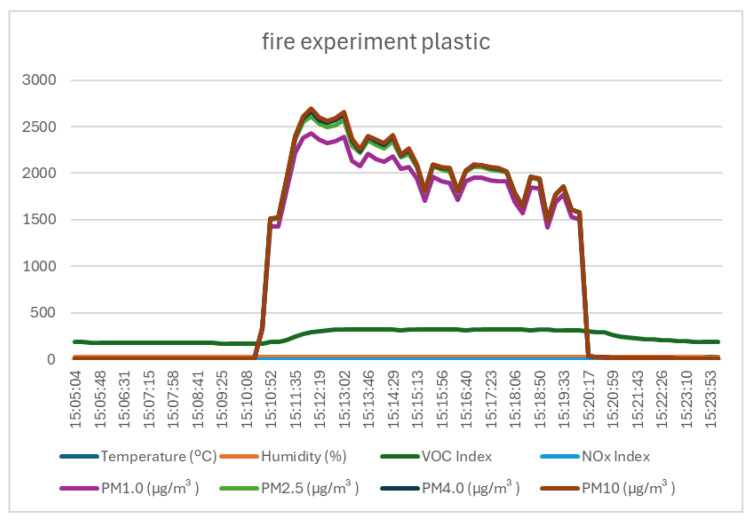
Graph of data recorded by monitoring system in case of plastic burning.

The third test used small pieces of plastic from various objects that are always found in a university laboratory: pieces of insulation from a UTP cable and an electrical cable, pieces of a computer keyboard, and pieces of the synthetic upholstery of a chair. The information collected by the Sensirion SEN55 sensor for this test was similar to the first two tests, with negligible variations in the parameters of temperature, ambient humidity, and NOx index, and significant variations in the VOC index (151 units) and airborne particles (variations of 2423 µg/m^3^ for PM1.0 and 2681 µg/m^3^ for PM10)—the test results are presented in Figure 8 and Table 6.

All three ignition tests identified a common behavior in the first minutes of the fire, with the small variations being characteristic of the materials burned and the way the materials burned. Because highly flammable materials that can give rise to an explosive fire were not tested, the first part of the ignition of the tested materials did not generate a large heat release (as evidenced by the temperature in the test chamber not having a significant variation), but they had a massive release of smoke and substances resulting from the incomplete combustion of the tested materials (as evidenced by the increase in volatile organic substances and particles). This model behavior recorded by the measurements performed will form the basis of the fire alarm-triggering mechanism presented in the next chapter of this paper.

The next two tests aimed to identify the behavior recorded for events that do not involve fire but can easily fool classic fire detection sensors: steam and dust from sources other than smoke emanating during combustion. Both phenomena generate fine particles (fine water particles or other types) that can be easily identified as smoke. The advantage of using a sensor that can measure multiple environmental parameters is that the fire alarm can exclude false-positive situations, such as a dust storm or a flood from the building’s heating system.

### 4.4. Test 4—Description, Execution, and Data

**Figure 9:**
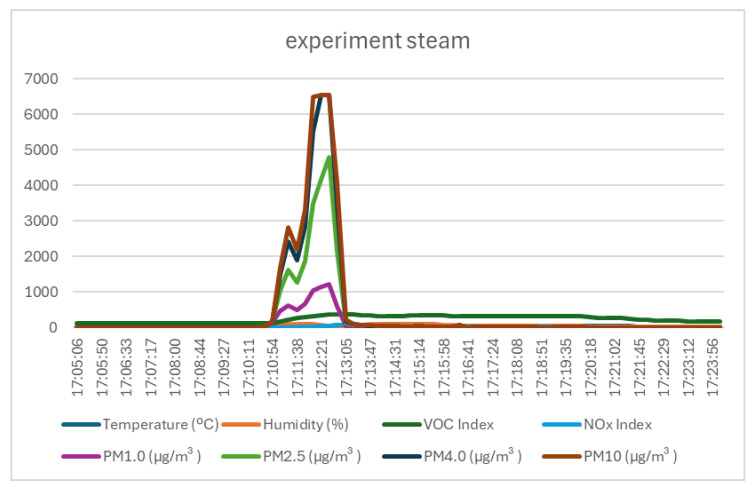
Graph of data recorded by the monitoring system in the experiment with steam.

The fourth test involved generating steam using an electric mug in the test chamber where the test sensor was located. The test showed, as can be seen in Figure 9 and Table 7, that the temperature and humidity increased very rapidly beyond the normal operating range of the sensor, reaching 73 °C and 100% humidity. The datasheet [20] for the Sensirion SEN55 sensor specifies that the maximum operating conditions are 50 °C and 90% relative humidity. Operating outside the normal conditions indicated by the manufacturer means that the other environmental parameters measured by the sensor (VOC index, NOx index, PM1.0, PM2.5, PM4.0, and PM10) cannot be considered, and provides a clear way to identify the tested event.

### 4.5. Test 5—Description, Execution, and Data

**Figure 10:**
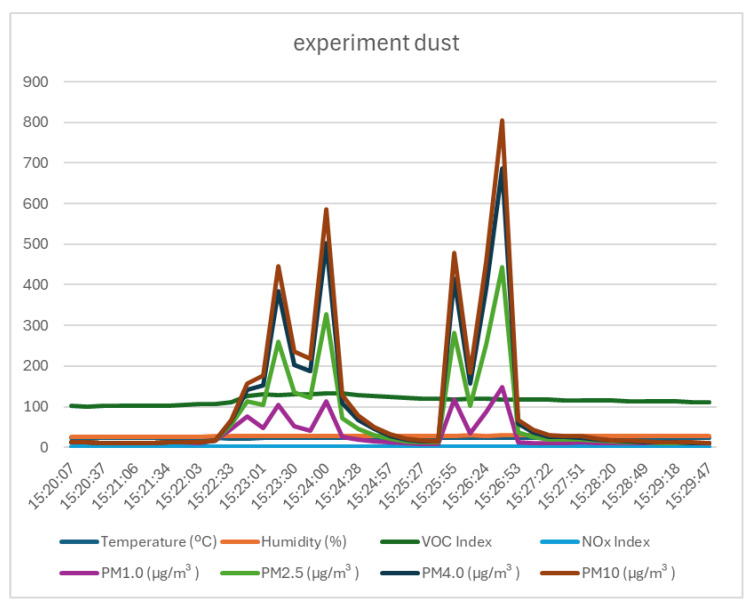
Graph of data recorded by the monitoring system in the experiment with dust.

The last test attempted to produce a large amount of common dust (dust normally present in the atmosphere) by reversing the suction direction of a household vacuum cleaner full of dirt inside the test enclosure. Even though the practical effect of the test was quite dirty, the values measured by the test sensor were relatively modest (as can be seen in Figure 10 and Table 8, the increase in the concentration of fine particles was a maximum of 795 µg/m^3^ in the case of PM10) compared to the values measured in the case of smoke generated by a fire. This test demonstrated that even if we are talking about an abnormal concentration of natural dust, this phenomenon will not be confused with that generated by a fire if a sensor is used that is capable of accurately indicating the concentration of dust in the atmosphere, rather than one that just detects the presence of particles in the air.

### 4.6. Statistical Determination of the Variation Threshold for Environmental Parameters Under Normal Operating Conditions

To better understand the values recorded following the 5 tests presented above, it was necessary to analyze the environmental parameters under normal conditions over a longer period of time. The data recorded over three months (between 1 April 2025 and 30 June 2025) by the AIR811_PR205 sensor in the PR205 laboratory were used. The measurements were recorded at intervals of 5 min. During the analyzed period, the laboratory operated normally without any incident likely to influence the recorded data (any incident involving fire or smoke emissions). The primary statistical analysis of the recorded data is presented in Table 9.

Following the statistical analysis, it can be seen that the only measured parameter that had a strong variation was the VOC index, which covered the entire measurement range (0–500). This indicates that an instantaneous value for this parameter can have extreme (maximum) values even in the context of regular operations, and therefore cannot be used alone as a trigger for a special situation. For the parameters indicating dust concentrations, maximum values around 70 µg/m^3^ were observed. The maximum value was well below the values recorded in the previous tests and represents the key parameters for the proposed detection mechanism. However, as the dust experiment highlighted, these parameters cannot be used alone either. The combined use of the dust concentration value in the air and the VOC index ensures a sensor fusion that can eliminate false detection cases in the case of fire-type events.

To validate the statistical threshold for identifying the values that would trigger the alarm mechanism, a method based on the Z-Score (3-Sigma method) was used. First, the moving average was constructed over a 24 h window, and every 5 min the average of the values from the last 24 h was calculated. The statistical analysis of the series of moving averages is presented in Table 10. The variation ranges of all the measured parameters could be strongly decreased by diminishing the influence of the maximum values in the measurements.

Based on the calculated average series, the difference between the instantaneous values and the average value was calculated. A series of values was obtained that indicate the usual variation mode under normal conditions. For this series of difference values, the mean (*μ_diff*) and the standard deviation (*σ_diff*) were calculated to evaluate the fluctuations generated normally—the calculated values are presented in Table 11.

The threshold for detecting situations indicating an abnormal variation in a measured value is defined as a multiple of the standard deviation, according to Formula (1). Using a factor of 3 ensures that 99.7% of normal fluctuations will not be flagged as anomalies, reducing false alarms. The detection threshold values are calculated in Table 10.

(1) Treshold=μ_diff+(3×σ_diff)

The threshold values calculated following this statistical analysis, together with all the observations made following the 5 tests, form the basis for the implementation of the alarm algorithm presented below.

### 4.7. Discussion of Tests Performed

The five tests conducted in this study aimed to identify the pattern required to detect a fire in a laboratory room at an early stage and to distinguish between a false-positive event and a real fire. The tests used a low-cost IoT indoor air quality monitoring system that had a single sensor system not specialized for fire detection. The Sensirion SEN55 sensor is a low-cost sensor with a high degree of integration of indoor air quality measurement functionalities, but is not designed for fire detection. Despite this, the tests performed proved that the diverse functionality of measuring several environmental parameters can be successfully used to identify fire outbreak events in a university laboratory.

In terms of the recorded parameters, the identified special behavior patterns are divided into the following classes:

- The sensor is in abnormal operating conditions, a pattern identified following the steam test, when the temperature and humidity parameters exceed the normal operating conditions given by the manufacturer. This class of situations may indicate an event of a type other than a fire (for example, the rupture of a building heating pipe) or an advanced stage of a fire (a case that falls outside the current research area).
- The sensor indicates a dangerous situation for human health, but not a fire. In this case, the value of one of the air quality parameters is above normal values (as was the case for the dust concentration in the last test), but its increase is large only in relation to indoor air quality standards and not to the sensor’s measurement range (the test carried out indicated an increase of 150–700 µg/m^3^ in the dust concentration for the various particle sizes).
- Early detection situation of a fire, characterized by a strong increase in the dust concentration (over 2000 µg/m^3^ regardless of particle size PM1.0, PM2.5, PM4.0, or PM10) simultaneously with a strong increase in another air quality parameter (in the case of the present research, the VOC index increased by over 150 units, but it is possible that in the case of burning materials with higher calorific values or containing hydrocarbons, it could also be the NOx index).

The correct automatic detection of events is very important for a fire detection system, and the tests performed allow for the proposal of an efficient fire alarm mechanism at an IoT platform level; the mechanism is presented in the next section.

## 5. Fire Detection Assistance Components Based on IoT Monitoring System

The IoT air quality monitoring system presented in Section 3 of this paper is based on the open-source ThingsBoard Community Edition platform as the central IoT platform. The ThingsBoard platform offers advanced capabilities for managing IoT devices, saving and processing the data received from IoT devices, as well as for displaying data and interacting with a user. The tests performed and presented in Section 4 of this paper aimed to establish the criteria for recognizing fire-type events and to implement rapid ways of alerting the user and providing a geospatial presentation of the building in which it occurs to allow for planning the firefighting intervention. The current research aims to experimentally extend the functionality of the IoT platform presented in Section 3 with fire detection and intervention assistance functionalities. The functionalities described in the current section do not aim at this time to replace existing fire detection systems, but only to prove the possibility of experimentally extending the functionality of the existing indoor air quality monitoring system.

### 5.1. Alarm-Triggering Process

The first element required for adding the extended experimental functionality to fire detection is the processing and detection of special situations. For each new MQTT message containing valid data from any of the ten sensors involved in the current research (symbolically marked with the AIR811ss model, which indicates the use of the Sensirion SEN55 sensor), the difference between the average values of the last 24 h and the newly received value is calculated, resulting in a new value that is saved in the series of values associated with the sensor for which it is calculated under the name delta. This process is performed for the VOC index and the PM10 parameters. The tests described above showed that the variations in the VOC index and dust concentration parameters are the ones that allow for the categorical identification of fire-type events. Of all the sizes of dust particles, PM10 had the largest variation and so, to minimize the computational effort made by the central platform, only PM10 processing was chosen.

**Figure 11:**
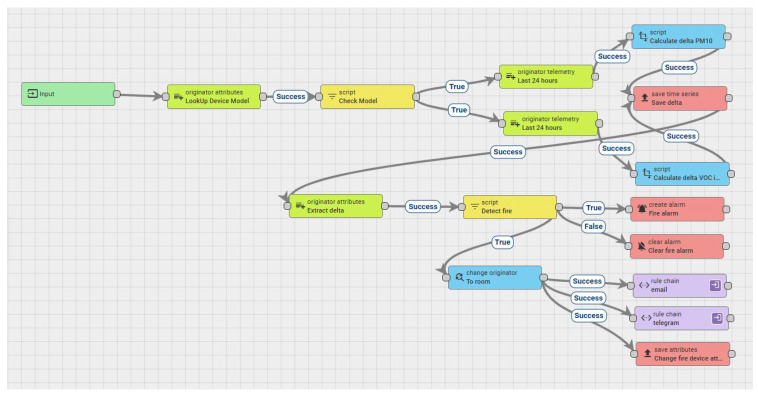
ThingsBoard rule chain for computing deltaVOC and deltaPM10 and triggering fire alarms.

The calculation of the differences between the last value and the average of the values from the last 24 h for the VOC index and PM10 parameters is conducted within a rule chain within the ThingsBoard platform, as shown in Figure 11. Equation (2) details the calculation formula for the two delta parameters (deltaVOC and deltaPM10). The difference is determined for the last measured value (*last value*) and the average of the values from the last 24 h; the delta value is evaluated without a sign.

(2) $$delta=\left|\frac{\sum_{last\;24\;h}parameter}{numbers\;of\;values}-last\;value\right|\;$$

**Figure 12:**
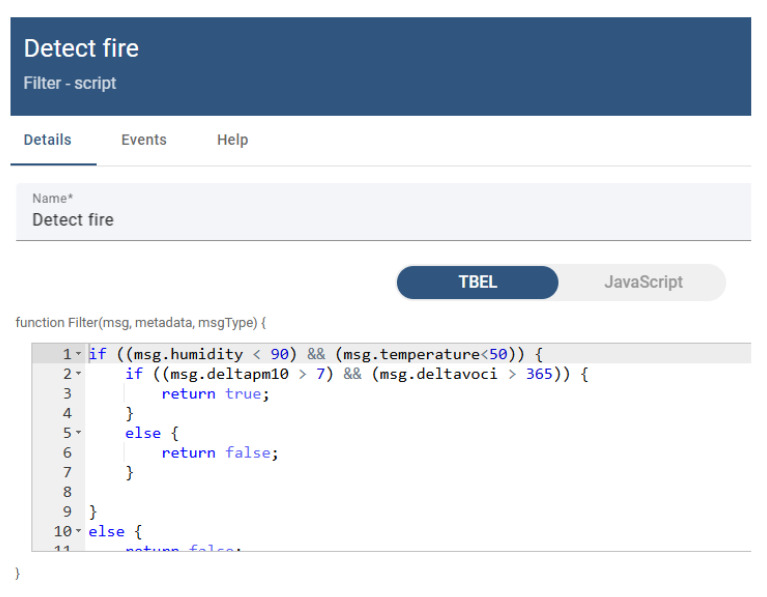
ThingsBoard “Detect fire” node—alarm-triggering condition. The trigger limits are hardcoded for better visualization of the condition.

Rule chains within the ThingsBoard platform allow for the processing of messages received from IoT devices, and the making of decisions and generating actions. In the case of our rule chain, the type of device that sends the data is checked to be an AIR811ss (the “Check Model” node), the values from the last 24 h for the VOC index and PM10 are extracted from the database, the average is determined and the difference between the last value and this average is calculated (the “Last 24 h” and “Calculate delta” nodes), and this value is saved in the database. The “Detect fire” node checks the operating condition in relation to the normal parameters in terms of temperature and humidity (humidity less than 90% and temperature less than 50 °C), and checks the delta values for the VOC index and PM10 (deltavoci greater than 465 and deltapm10 greater than 7), as shown in Figure 12.

**Figure 13:**
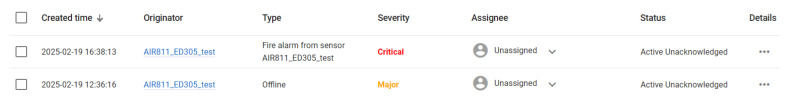
ThingsBoard alarms dashboard.

**Figure 14:**
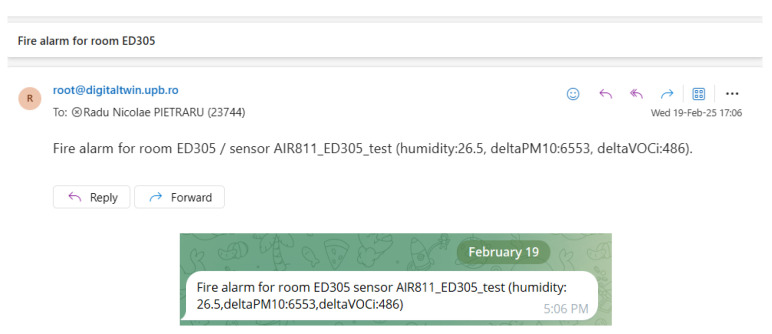
Fire alarms (up—email; bottom—telegram).

The alarm is triggered when both the normal operating conditions for the environmental parameters and increases in the values of the VOC index and PM10 are met, and the following actions are generated:

- An alarm is generated at the ThingsBoard platform level (Figure 13). This alarm is visible at the platform level and can be managed in a complex manner by assigning it to the person in charge, associating the event description, and tracking the resolution. This alarm has the role of alerting the people responsible for monitoring the building.
- Notifications are sent to the person in charge of the laboratory in which the alarm was triggered. At the ThingsBoard platform level, the IoT sensors belong to specific rooms, and each room has a defined person in charge. In the current implementation, alarms are sent via email and the Telegram platform (Figure 14), but the ThingsBoard platform also allows the implementation of alarms via SMS or at the ThingsBoard mobile application level. These alarms have the role of alerting the local people in charge.
- Activate the sound and light alarms on the hardware devices by changing the alarm state. The alarm hardware devices are IoT devices consisting of a NodeMCU development board, a siren, and a flash LED bar. These devices periodically check the status of a server parameter via WiFi and, depending on this, have an on or off status. These devices have the role of alarming the people in the immediate vicinity of the area where the fire was detected.

### 5.2. Geospatial Representation of the Space Where the Fire Starts

**Figure 15:**
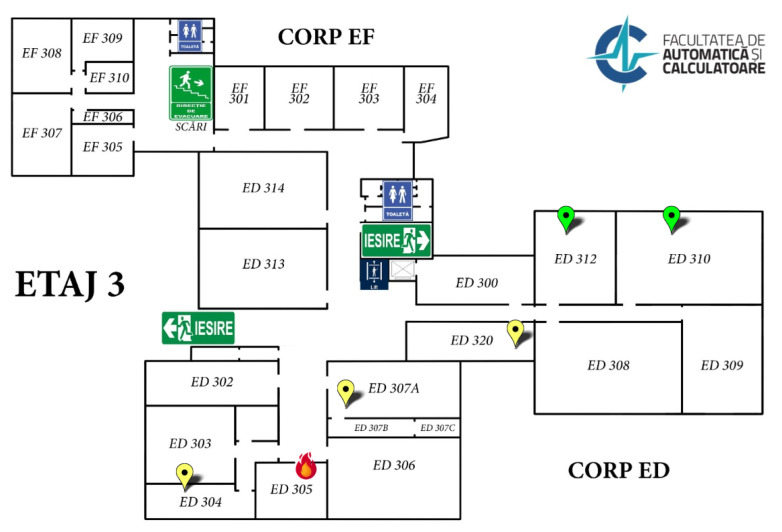
ThingsBoard dashboard for floor 3 of ED building—virtual sensor triggering example for Room ED305 (the flame symbol indicates a fire alarm, the green color of the sensors on the map indicates excellent air quality in the room, and the yellow color indicates good quality).

To test the alarm-triggering mechanism, a virtual IoT device—AIR811_ED305_test—was registered on the ThingsBoard platform for floor 3 of the ED building on campus. Sending data on behalf of this device was simulated using a MQTT mosquito client on a computer. The data from previously performed tests (both ignition and false-positive tests) were used as the test data. The testing aimed for correct alarm triggering only in cases of ignition and not for false positives (steam and dust). As can be seen in the previous figures (Figure 11 and Figure 12), the alarm was triggered correctly if the fire detection conditions were met. The dashboard associated with floor 3 of the ED building, where the virtual sensor was registered (room ED305 was chosen because there is no real IoT sensor in this room), also presented the correct fire situation (Figure 15).

**Figure 16:**
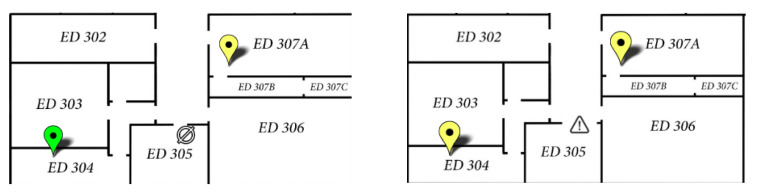
ThingsBoard dashboard for floor 3 of ED building—disconnected sensor (**left**) and malfunctioning sensor (**right**).

Before implementing the new fire warning mechanism, the representation of IoT air quality monitoring sensors was conducted through a pinpoint that changed color depending on the measurements of each sensor (green—excellent conditions; yellow—average conditions; orange—poor conditions; red—unhealthy conditions; detailed color scale in [18]). After adding the new mechanism, in order not to interfere with the color signaling already implemented, the graphical representation of the sensors on the building’s geospatial dashboard was changed in case of fire conditions, a disconnected sensor, or a malfunctioning sensor (Figure 14 shows the icon used for fire; Figure 16 shows the other two icons).

The integration of the new fire detection mechanism within the air quality monitoring platform allows for easy visualization of the geospatial situation in the event of a fire that spreads to multiple rooms or even floors of a building. After the initial alarms are issued, the fire teams can visualize which spaces are actually affected. The disconnected state of sensors can indicate the total compromise of the electrical networks and the data can indicate areas catastrophically affected by the fire. Areas that are not actually affected by the fire and are not flooded with smoke may still have dangerous air quality parameters, and may be areas that the intervention teams must evacuate as a priority. The indications from the IoT monitoring platform could prove to be extremely valuable by providing data from a building engulfed in flames through geospatial dashboards.

## 6. Conclusions and Future Developments

### 6.1. Conclusions

This paper aimed to present an experimental solution for the functional expansion of an IoT system for indoor air quality monitoring by adding a logical fire detection component in the buildings of a university campus; the IoT air quality monitoring system is presented in Section 3. The proposed solution did not aim to design sensor systems specific to fire detection, but rather to use IoT sensors already used for air quality measurement—the IoT sensors presented in Section 3. By performing the tests presented in Section 4, it was observed that situations involving the ignition of common materials currently found in laboratory rooms on the university campus led to severe changes in the air quality parameters, changes that could clearly identify a fire situation. Other common events encountered in the daily routine of activity within a teaching laboratory do not have similar effects and do not generate false-positive alarm situations. The identification of thresholds for detecting abnormal situations was achieved through a statistical method based on the Z-Score.

The experimental solution proposed in this paper does not intend to replace the classic fire detection systems in use, as it is not mature enough and is insufficiently tested for this, but it can influence the design of future generations of fire protection systems in smart campuses/smart buildings by demonstrating advanced IoT functionalities, such as multiple alarm methods, data fusion with IoT sensor systems, real-time geospatial representation of buildings, and providing advanced assistance to intervention teams in case of dangerous events. The present study proves that the integration of multiple acquisition and detection systems can be useful for improving the functioning of assistance systems in smart buildings; the integration of air quality monitoring systems and fire detection systems is just an example suggested by the current study, but sensor fusion could go beyond the smart building field and be extended to assistive medicine or to monitoring outdoor environmental parameters and climate change.

The idea of using air quality sensors for fire detection is not new; Section 2 of the paper presents several scientific studies that have addressed this issue. The results presented in this paper detail the successful implementation of an experimental fire detection method within an IoT platform for air quality monitoring on a university campus that has been in use for over three years; it is a functional monitoring platform that has received the attention of and is used by a large community of researchers at our university. The parallel operation of the experimental fire detection solution within the IoT monitoring platform with the classic fire detection system in the university’s campus buildings may reveal important elements for the fusion of both systems in the future. The proposed experimental solution is a good starting point for the scientific research necessary for the design of IoT assistance systems in the smart buildings of the future.

Summarizing the original results of the current research, the following can be highlighted:

- The tests performed and presented in Section 4 of the paper prove the capability of an IoT IAQ monitoring network to sense the beginning signs of a fire, which proves the viability of exploring such a solution.
- Adding fire alarm-triggering mechanisms to an IoT monitoring platform in a smart building increases the diversity of fire alarm mechanisms for both building occupants and building management and intervention teams, which are presented in Section 5.1.
- The geospatial representation offered by the cloud platform through a specific dashboard provides a clear picture of the location and status of neighboring premises, useful for the efficient and safe intervention of intervention teams; these elements are presented in Section 5.2.

Even if the current research cannot be considered a complete alternative to current fire detection systems, it can be concluded that it possesses sufficient evidence and advantages to be considered a promising direction for the development of a new generation of safety systems in smart buildings.

### 6.2. Future Development Directions

A scientific aspect not addressed by the current research, but very important for the development of high-performance fire protection systems, is the analysis of the air composition in the moment preceding the start of a fire—the moment before the fire ignites. Studies such as [21] have proven the possibility of designing an early detection system that would allow for an ultra-fast intervention in the event of the occurrence of fire-starting conditions. This is an important future research direction based on the present study.

Based on our promising results, future research should focus on two critical directions, as also highlighted in the literature review. First, advanced statistical models need to be developed to distinguish fire anomalies from background fluctuations and to establish dynamic detection thresholds. Second, these models need to be validated using more complex experimental scenarios that simulate the heterogeneity of real fires.

One aspect that can be considered weak in the present research is the use of an air quality sensor that does not measure the relevant parameters in the event of a fire, such as CO or CO_2_. IoT sensors for air quality monitoring have been designed with the greatest possible simplicity in mind (only two components: a programmable circuit with network connectivity and a single sensor) and a best price/functionality ratio. The future development of integrated measurement elements (such as the availability this year of the Sensirion SEN6x [22] sensor generation) is likely to help improve this aspect. The way to connect IoT sensors to a network is another aspect that could be the subject of future improvement analyses. The current proposed solution uses the IEEE 802.11 [23] campus infrastructure, which has a vulnerability. In the event of a fire, it could cause the total or partial failure of the electrical network and the data network on campus. Redesigning IoT monitoring systems to operate on their own power sources (batteries) and to utilize fail-safe communication networks (such as mesh networks or LoRaWAN) are certainly possible future directions for improvement.

To be considered as a fire detection system, the proposed experimental solution requires mandatory testing in an accredited metrological laboratory, as well as a thorough verification of compliance with the legal requirements in force. The current study could not achieve these mandatory elements to be able to claim that the proposed experimental solution is a legal fire detection system. This effort is only a desideratum at the current time and all the elements presented in this paper must be treated as such.

## Figures and Tables

**Table 1:** Relevant studies.

The Study	Detection System	Monitoring Parameters	Decision Criteria	Connectivity Wireless Sensors	IOT Platform	Map/Intervention	Generate Alarm Events
[9]	WiFi LORA 32 sensors and board	Grove gas sensor MQ2	Unspecified	LoRaWAN, WiFi	Unspecified	No	No
[10]	Sensors network and microcontroller (ESP32)	PM, VOCs, CO, H2, CO_2_, UV photon, temperature, and relative air humidity GGS 6530 T gas sensor	Flamingcombustion, thresholds	WiFi using the MQTT protocol, Raspberry Pi	Grafana dashboard	No	No
[11]	Microcontroller Arduino Board	Humidity, temperature, MQ139, TVOCs, and eCO2 sensors	Flaming thresholds	LoRaWAN, WiFi	The Thing network cloud server	No	No
[12]	System on a chip, TI-CC2540 and TI-CC2541	Smoke and temperature sensors	False/true	Bluetooth	FireSense device	Yes	Yes
[13]	Microcontroller Arduino Uno	Temperature, humidity, gas, smoke, and flame sensors	Flame, presence of gas, thresholds	Unspecified	Cloud API	No	No
[14]	Processing hub-home sink	Temperature, smoke, and gas sensors	Thresholds	ZigBee protocol, GSM	None	Yes	Yes
[15]	Arduino small-scalemicrocontroller and NodeMCU module	Temperature, smoke, flame, and LDR sensors	Thresholds	WiFi	Blynk Cloud	Yes	Yes
[16]	ESP8266 WiFi-enabled NodeMCUmicrocontroller	DHT11 temperature and humidity sensor, MQ135 air quality sensor, and flame sensors	Thresholds	WiFi	Blynk application (an IoT platform) with Thingspeak cloud storage	No	No
Our study	Own air quality monitoring sensors based on NodeMCU ESP8266 and SEN55—low cost and easy to deploy as proven in [17]	VOCs NOx PM1.0, PM2.5, PM4.0,and PM10 temperature and humidity sensor	Sensor fusion—multiple trigger conditions	WiFi	ThingsBoard IoT Cloud Platform	Yes/can be used to organize the intervention of firefighters	Yes—email, mobile phone alarm, web Platform

**Table 2:** Sensirion SEN55 technical details related to the measured parameters (information extracted from the original manufacturer’s datasheet).

Parameter		Measured Parameter	Measurement Range	Precision
Mass concentration size range	PM1.0	0.3–1 μm	0–1000 μg/m^3^	±10%
	PM2.5	0.3–2.5 μm	0–1000 μg/m^3^	±10%
	PM4	0.3–4 μm	0–1000 μg/m^3^	±10%
	PM10	0.3–10 μm	0–1000 μg/m^3^	±10%
VOC Index		Index	1–500	±15%
NOx Index		Index	1–500	±15%
Temperature		Temperature	−10–50 °C	±0.45 °C
Humidity		Humidity	0–90%RH	±4.5%RH

**Table 3:** Test description.

Test Name	Material
Test 1	stickers
Test 2	wooden matches
Test 3	plastic
Test 4	steam (using an electric teapot)
Test 5	dust (by reversing the ventilation direction of a dirty vacuum cleaner)

**Table 4:** Statistical variation in values recorded during entire sticker burning experiment [13:05–13:25] (AVG—average; MED—median value; STD—standard deviation; MAX—maximum value; MIN—minimum value; Variation = MAX − MIN).

	AVG	MED	STD	MAX	MIN	Variation
Temperature	25.14	25.14	0.05	25.20	25.07	0.14
Humidity	25.92	26.26	1.14	27.03	23.88	3.15
VOC Index	122.08	102.00	69.33	395.00	31.00	364.00
NOx Index	1.38	1.00	0.49	2.00	1.00	1.00
PM1.0	1215.44	78.60	1266.68	2748.10	7.70	2740.40
PM2.5	1577.46	94.45	1652.65	3675.30	8.10	3667.20
PM4.0	1831.31	104.55	1925.61	4352.10	8.10	4344.00
PM10	1953.77	109.40	2057.51	4678.60	8.10	4670.50

**Table 5:** Statistical variation in values recorded during entire wooden matches burning experiment [13:45–14:05] (AVG—average; MED—median value; STD—standard deviation; MAX—maximum value; MIN—minimum value; Variation = MAX − MIN).

	AVG	MED	STD	MAX	MIN	Variation
Temperature	24.62	24.68	0.11	24.73	24.42	0.31
Humidity	24.66	25.48	1.62	26.67	22.64	4.03
VOC Index	337.81	434.50	160.61	499.00	64.00	435.00
NOx Index	1.00	1.00	0.00	1.00	1.00	0.00
PM1.0	3014.20	5548.40	2841.21	6214.40	6.10	6208.30
PM2.5	3420.66	6553.40	3200.74	6553.40	6.40	6547.00
PM4.0	3437.68	6553.40	3190.53	6553.40	6.40	6547.00
PM10	3445.89	6553.40	3186.53	6553.40	6.40	6547.00

**Table 6:** Statistical variation in values recorded during entire plastic burning experiment [15:05–15:25] (AVG—average; MED—median value; STD—standard deviation; MAX—maximum value; MIN—minimum value; Variation = MAX − MIN).

	AVG	MED	STD	MAX	MIN	Variation
Temperature	23.50	23.66	0.33	23.85	23.06	0.79
Humidity	24.93	24.58	1.47	26.82	22.57	4.25
VOC Index	247.83	234.00	64.32	323.00	172.00	151.00
NOx Index	1.00	1.00	0.00	1.00	1.00	0.00
PM1.0	921.81	23.90	984.81	2432.30	8.80	2423.50
PM2.5	978.79	25.00	1047.88	2613.20	9.30	2603.90
PM4.0	988.91	25.00	1060.63	2665.50	9.40	2656.10
PM10	993.80	25.00	1066.82	2690.70	9.40	2681.30

**Table 7:** Statistical variation in the values recorded during the entire steam experiment [17:05–17:25] (AVG—average; MED—median value; STD—standard deviation; MAX—maximum value; MIN—minimum value; Variation = MAX − MIN).

	AVG	MED	STD	MAX	MIN	Variation
Temperature	29.66	27.61	9.30	73.01	14.59	58.43
Humidity	43.49	26.32	27.27	100.00	20.12	79.88
VOC Index	232.00	253.00	95.77	371.00	113.00	258.00
NOx Index	11.61	4.00	14.77	62.00	1.00	61.00
PM1.0	88.07	6.50	245.69	1218.20	4.20	1214.00
PM2.5	260.58	6.80	858.83	4789.60	4.40	4785.20
PM4.0	386.03	6.80	1286.24	6553.40	4.40	6549.00
PM10	422.48	6.80	1381.30	6553.40	4.40	6549.00

**Table 8:** Statistical variation in the values recorded during the entire dust experiment [15:20–15:30] (AVG—average; MED—median value; STD—standard deviation; MAX—maximum value; MIN—minimum value; Variation = MAX − MIN).

	AVG	MED	STD	MAX	MIN	Variation
Temperature	23.08	23.10	0.07	23.16	22.98	0.18
Humidity	27.97	28.18	0.93	29.42	26.20	3.22
VOC Index	116.12	116.00	9.56	133.00	101.00	32.00
NOx Index	1.00	1.00	0.00	1.00	1.00	0.00
PM1.0	29.23	11.75	35.32	147.30	9.20	138.10
PM2.5	65.85	17.20	101.25	444.50	9.90	434.60
PM4.0	95.36	19.95	155.85	687.60	9.90	677.70
PM10	109.59	21.90	182.22	804.90	9.90	795.00

**Table 9:** Statistical variation in values recorded during three months of normal operation—instantaneous values (AVG—average; MED—median value; STD—standard deviation; MAX—maximum value; MIN—minimum value; Variation = MAX − MIN).

	AVG	MED	Mode	STD	MAX	MIN	Variation
Temperature	24.11	24.29	24.39	1.28	30.54	20.49	10.06
Humidity	44.36	43.96	46.08	8.93	69.23	20.18	49.05
VOC Index	147.47	110.00	101.00	121.43	500.00	0.00	500.00
NOx Index	1.84	1.00	1.00	1.64	31.00	0.00	31.00
PM1.0	4.06	3.60	2.40	2.45	66.10	0.20	65.90
PM2.5	4.25	3.70	2.50	2.57	69.70	0.20	69.50
PM4.0	4.25	3.70	2.50	2.57	70.00	0.20	69.80
PM10	4.25	3.70	2.50	2.57	70.10	0.20	69.90

**Table 10:** Statistical variation in values recorded during three months of normal operation—24 h average (AVG—average; MED—median value; STD—standard deviation; MAX—maximum value; MIN—minimum value; Variation = MAX − MIN).

	AVG	MED	Mode	STD	MAX	MIN	Variation
Temperature	24.12	24.41	25.22	1.03	25.40	21.64	3.76
Humidity	44.29	44.52	60.62	7.42	60.85	31.79	29.06
VOC Index	146.98	143.70	133.72	21.47	203.75	106.47	97.28
NOx Index	1.84	1.78	1.60	0.42	3.12	1.12	2.01
PM1.0	4.05	3.65	3.05	1.61	7.90	1.87	6.03
PM2.5	4.24	3.83	3.20	1.69	8.29	1.97	6.32
PM4.0	4.24	3.83	3.79	1.69	8.29	1.96	6.33
PM10	4.25	3.83	3.20	1.69	8.29	1.96	6.33

**Table 11:** Values calculated for the series of differences between the instantaneous values and the 24 h moving average (*μ_diff*—average of the series of differences between the 24 h moving average and the instantaneous value; *σ_diff*—standard deviation of the series of differences between the 24 h moving average and the instantaneous value; *Threshold*—threshold value calculated using Formula (1)).

	*μ_diff*	*σ_diff*	*Threshold*
Temperature	0.0887	0.8526	2.6465
Humidity	0.6342	0.8526	19.2730
VOC Index	0.9925	121.6367	365.9025
NOx Index	0.0089	1.6530	4.9500
PM1.0	0.0171	2.0323	6.0798
PM2.5	0.0179	2.1328	6.3804
PM4.0	0.0180	2.1340	6.3840
PM10	0.0180	2.1345	6.3855

## Data Availability

The original contributions presented in this study are included in the article. Further inquiries can be directed to the corresponding author.

## References

[1] McNay J. (2022). A Guide to Fire and Gas Detection Design in Hazardous Industries.

[2] Hess-Kosa K. (2018). Indoor Air Quality: The Latest Sampling and Analytical Methods.

[3] Khan F., Xu Z., Sun J., Khan F.M., Ahmed A., Zhao Y. (2022). Recent Advances in Sensors for Fire Detection. Sensors.

[4] Woolley T. (2016). Building Materials, Health and Indoor Air Quality: No Breathing Space?.

[5] Kanagamalliga S., Aarthi Radha T.S., Vengadakrishnan S., Sridhar R., Adinkrah-Appiah K., Rajalingam S., Nanda U., Tripathy A.K., Sahoo J.P., Sarkar M., Li K.C. (2024). Harnessing IoT-Powered Fire Detection Systems for Enhanced Security. Advances in Distributed Computing and Machine Learning.

[6] Fonollosa J., Solórzano A., Marco S. (2018). Chemical Sensor Systems and Associated Algorithms for Fire Detection: A Review. Sensors.

[7] Vaegae N.K., Annepu V., Bagadi K., Dahan F. (2024). Multisensor Fuzzy Logic Approach for Enhanced Fire Detection in Smart Cities. J. Optim..

[8] Çetin A.E., Merci B., Günay O., Töreyin B.U., Verstockt S. (2016). Methods and Techniques for Fire Detection.

[9] Safi A., Ahmad Z., Jehangiri A.I., Latip R., Zaman S.K.U., Khan M.A., Ghoniem R.M. (2022). A Fault Tolerant Surveillance System for Fire Detection and Prevention Using LoRaWAN in Smart Buildings. Sensors.

[10] Vorwerk P., Kellter J., Müller S., Krause U. (2023). Distance-Based Analysis of Early Fire Indicators on a New Indoor Laboratory Dataset with Distributed Multi-Sensor Nodes. Fire.

[11] Nazir A., Mosleh H., Takruri M., Jallad A.-H., Alhebsi H. (2022). Early Fire Detection: A New Indoor Laboratory Dataset and Data Distribution Analysis. Fire.

[12] Cheng M.Y., Chiu K.C., Hsieh Y.M., Yang I.T., Chou J.S., Wu Y.W. (2017). BIM integrated smart monitoring technique for building fire prevention and disaster relief. Autom. Constr..

[13] Lule E., Mikeka C., Ngenzi A., Mukanyiligira D. (2020). Design of an IoT-Based Fuzzy Approximation Prediction Model for Early Fire Detection to Aid Public Safety and Control in the Local Urban Markets. Symmetry.

[14] Saeed F., Paul A., Rehman A., Hong W.H., Seo H. (2018). IoT-Based Intelligent Modeling of Smart Home Environment for Fire Prevention and Safety. J. Sens. Actuator Netw..

[15] Saini M.L., Kumar R., Sati D.C., Bharti T. Fire Monitoring and Prevention System Based on the Severity of Fire Using NodeMCU and Cloud. Proceedings of the 2024 International Conference on Intelligent and Innovative Technologies in Computing, Electrical and Electronics (IITCEE).

[16] Kharade M., Katangle S., Kale G.M., Deosarkar S.B., Nalbalwar S.L. A NodeMCU based Fire Safety and Air Quality Monitoring Device. Proceedings of the 2020 International Conference for Emerging Technology (INCET).

[17] Pietraru R.N., Crăciun R.-A., Merezeanu D.-M. Rapid Deployment of Low-Cost Wireless Monitoring Solution for Smart Building. Proceedings of the 2024 International Conference Automatics and Informatics (ICAI).

[18] Bidilă T., Pietraru R.N., Ionita A.D., Olteanu A. Monitor Indoor Air Quality to Assess the Risk of COVID-19 Transmission. Proceedings of the 2021 23rd International Conference on Control Systems and Computer Science (CSCS).

[19] Pietraru R.N., Olteanu A., Velicu A.Ş., Craciun R.-A. Healing IoT Data for Indoor Air Quality Using Artificial Intelligence. Proceedings of the 2024 E-Health and Bioengineering Conference (EHB).

[20] Datasheet SEN5x Environmental Sensor Node for HVAC and Air Quality Applications. https://sensirion.com/media/documents/6791EFA0/62A1F68F/Sensirion_Datasheet_Environmental_Node_SEN5x.pdf.

[21] Solórzano A., Eichmann J., Fernández L., Ziems B., Jiménez-Soto J.M., Marco S., Fonollosa J. (2022). Early fire detection based on gas sensor arrays: Multivariate calibration and validation. Sens. Actuators B Chem..

[22] Simplifying Indoor Air Quality Sensing. https://sensirion.com/sen6x-air-quality-sensor-platform.

[23] (2025). IEEE Standard for Information Technology—Telecommunications and Information Exchange between Systems Local and Metropolitan Area Networks—Specific Requirements Part 11: Wireless LAN Medium Access Control (MAC) and Physical Layer (PHY) Specifications.

